# 

**Published:** 1866-03

**Authors:** 


### CONTENTS.

ORIGINAL COMMUNICATIONS.

Dental Materia Medica ... 101
The duty of the Dentist to his Profession, his Patient and to himself ... 105
Catarrhal Inflammation of the Antrum ... 114
An Address by Dr. Field ... 118
Digestion ... 121
Splitting Forceps ... 124
Address to Michigan Dental Association ... 126
Wanderings of Pus ... 129

PROCEEDINGS OF SOCIETIES.

Eleventh Annual Meeting of the Michigan Dental Association ... 133

EDITORIAL.

Stuffing Teeth ... 141
Ohio Dental College Association ... 143
Michigan Dental Association ... 144
Commencement Exercises ... 145
Mississippi Valley Dental Association ... 146
Journalistic ... 146
Hymenial ... 146

